# An endoscopic presentation of metastasis to the gastrointestinal tract: the volcano lesion

**DOI:** 10.1016/j.igie.2025.09.011

**Published:** 2025-09-23

**Authors:** Ashwariya Ohri, Davekaran Buttar, Mayank Goyal, Preeyati Chopra, Raseen Tariq, Andrew C. Storm

**Affiliations:** Division of Gastroenterology and Hepatology, Mayo Clinic, Rochester, Minnesota, USA

We present the case of a 93-year-old man status post wide local excision with negative margins for malignant melanoma performed 4 years before presentation and status post intralesional interleukin-2 treatment for biopsy-proven in-transit metastases of the left arm. A positron emission tomography–computed tomography scan performed 1 year before presentation was negative for distant metastasis. He presented with a history of dark stools for 3 weeks with down-trending hemoglobin. Chest/abdomen/pelvis computed tomography demonstrated an overall picture of metastatic malignancy of unclear etiology, with left upper quadrant omental nodularity and pelvic peritoneal enhancement. On esophagogastroduodenoscopy and colonoscopy, multiple lesions, elevated and ulcerated at the apex, characteristic of “volcano lesions” were noted in the stomach (Fig. 1A), duodenum (Fig. 1B), and colon (Fig. 1C1 and C2). These lesions should alert the endoscopist to possible metastasis, and further histologic evidence from the biopsies taken demonstrated neoplastic cells in the colon and the stomach (Fig. 1D1) that were strongly and diffusely positive for melanoma-associated antigen recognized by T cells 1 (MelanA [or MART 1]), confirming the diagnosis (Fig. 1D2). This case illustrates an unusual endoscopic presentation of extensive metastatic melanoma in the gastrointestinal tract in the form of volcano lesions.

**Figure 1:**
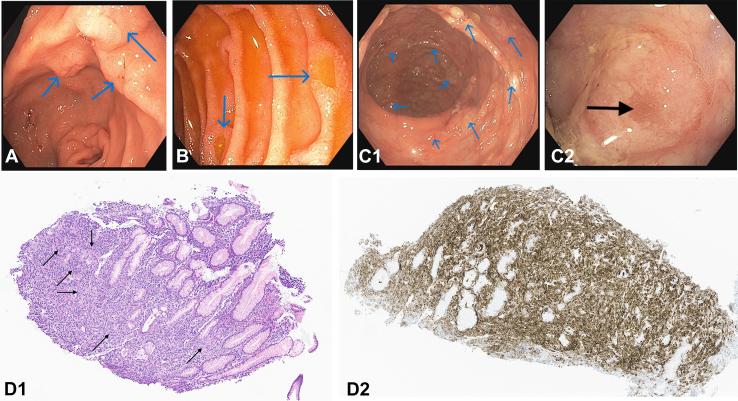
**A,** Endoscopic view of the stomach showing multiple elevated lesions with central ulceration (*arrows*) resembling “volcano-like” morphology. **B,** Endoscopic image of the duodenum with multiple unpigmented lesions (*arrows*) with characteristic volcano-like appearance. **C1,** Endoscopic view of the colon showing the multiple ulcerated and polypoid lesions (*arrows*); (**C2**) close-up view highlighting the characteristic “volcano” lesion, demonstrating peripheral elevation with central ulceration (*arrow*) in the colon. **D1,** Hematoxylin and eosin, original magnification ×200, showed pleomorphic and hyperchromatic melanoma cells forming tumor nests (*arrows*) in the mucosa and submucosa of the stomach (scattered throughout with interspersed gastric glands); (**D2**) hematoxylin and eosin, original magnification ×200, immunohistochemical staining demonstrating strong positivity for melanoma-associated antigen recognized by T cells 1 (MelanA [or MART-1], diffuse brown staining), confirming melanocytic origin.

## Patient Consent

Written informed consent was obtained from the patient for publication of clinical images, with effort made to protect patient identity and maintain confidentiality.

## Disclosure

The following author disclosed financial relationships: A. C. Storm: Research funding from Apollo Endosurgery, Boston Scientific, Endogenex, Endo-TAGSS, Enterasense, EnVision, MGI Medical, OnePass, and SofTac; consultant for Boston Scientific, Cook, Endo-TAGSS, Fuji, Sotelix, Steris, GI Dynamics, Intuitive, Lean Medical, Medtronic, Microtech, Olympus, and Qaelon. All other authors disclosed no financial relationships.

